# Morphological variability and genetic structure of Miscanthus sinensis (Poaceae) cultivated in the forest-steppe of Western Siberia

**DOI:** 10.18699/vjgb-25-24

**Published:** 2025-04

**Authors:** O.V. Dorogina, I.N. Kuban, G.A. Zueva, E.V. Zhmud, O.Yu. Vasilyeva

**Affiliations:** Central Siberian Botanical Garden of the Siberian Branch of the Russian Academy of Sciences, Novosibirsk, Russia Novosibirsk State University, Novosibirsk, Russia; Central Siberian Botanical Garden of the Siberian Branch of the Russian Academy of Sciences, Novosibirsk, Russia; Central Siberian Botanical Garden of the Siberian Branch of the Russian Academy of Sciences, Novosibirsk, Russia; Central Siberian Botanical Garden of the Siberian Branch of the Russian Academy of Sciences, Novosibirsk, Russia; Central Siberian Botanical Garden of the Siberian Branch of the Russian Academy of Sciences, Novosibirsk, Russia

**Keywords:** quality of seeds, decorative form of Miscanthus sinensis, ISSR markers, S1 and S2 selected forms, G1 and G2 generations, certification, качество семян, декоративная форма Miscanthus sinensis, ISSR-маркеры, отобранные формы S1 и S2, репродуктивные поколения G1 и G2, паспортизация

## Abstract

Miscanthus sinensis Andersson (Poaceae) grows in monsoon climate. For this reason, when cultured under the conditions of a short growing season of Western Siberia, full-fledged seeds do not have time to form. We have studied a large number of specimens of this species from Primorsky Krai in the collection of the Central Siberian Botanical Garden, SB RAS. Using these samples, it was possible for the first time to select forms that produce high-quality mature seeds in local conditions during a short growing season, possibly due to spontaneous hybridization of early flowering forms. We obtained the first and second (G1 and G2) generations from these seeds and checked for hybrids. The aim of this study is selection, biomorphological characterization of early flowering ornamental forms of M. sinensis and analysis of genetic polymorphism of the selected forms (S1, S2) and the obtained G1 and G2 generations using ISSR markers. Under the conditions of introduction, the selected samples of M. sinensis were characterized by complex resistance, high decorativeness, reached the ontogenetic state of mature generative plants and differed from other samples in the collection by early flowering and the formation of full-fledged seeds. Thus, the forms of M. sinensis we selected are promising for landscape design and breeding. When studying the genetic structure of G1, G2 and two generations of the sample using ISSR markers, three effective stable unique PCR fragments were identified. A study of the genetic variability of the resulting G1 generation showed complete uniformity of genotypes. In the G2 generation, variability was observed, and we found five sets of genotypes, which were also confirmed in the dendrogram. As a result, unique molecular polymorphic fragments were identified. Their length was 300–3000 bp, and the genetic formula for certification of M. sinensis was compiled.

## Abstract (ru)

Miscanthus sinensis Andersson (Poaceae), как и все другие виды рода Miscanthus, является растением муссонного климата, поэтому в суровых условиях лесостепи Западной Сибири не успевает сформировать жизнеспособные семена. При изучении большого числа образцов этого вида из коллекционного генофонда Центрального сибирского ботанического сада СО РАН, привезенных из различных районов Приморского края, впервые удалось отобрать две формы, S1 и S2 (selected), образующие в местных условиях в течение короткого вегетационного периода качественные зрелые семена, из которых были получены первое и второе, G1 и G2 (generation), репродуктивные поколения. Цель настоящего исследования – отбор, биоморфологическая характеристика раноцветущих декоративных форм M. sinensis и анализ генетического полиморфизма отобранных форм (S1, S2) и полученных поколений (G1, G2) с помощью маркеров ISSR. В условиях интродукции отобранные образцы M. sinensis характеризовались комплексной устойчивостью к болезням и вредителям, высокой декоративностью, ранними сроками цветения и образованием полноценных семян. При изучении генетического полиморфизма с помощью маркеров ISSR у родительских образцов S1 и S2 обнаружено от одного до трех уникальных ПЦР-фрагментов. Исследование генетической изменчивости в поколении G1 показало полное единообразие генотипов. В поколении G2 наблюдалась изменчивость, при этом нами найдено пять вариантов генотипов. В результате выявлены уникальные полиморфные фрагменты, длина которых варьировала от 300 до 3000 п. н.; с их помощью составлена генетическая формула для паспортизации отобранных нами форм M. sinensis. Эти формы характеризуются высокими репродуктивными способностями в условиях континентального климата, перспективны для селекции, а, благодаря своим биоморфологическим особенностям, – и для ландшафтного дизайна.

## Introduction

The genus Miscanthus Andersson (fan grass) belongs to the subtribe Saccharinae, tribe Andropogoneae, family Poaceae. Fan grasses are perennial herbaceous plants. They are characterized by the C4 type of photosynthesis, high biomass productivity and relative unpretentiousness. These features allow them to be recommended as a promising bioenergy crop (Nie et al., 2014). The issues of biology and production process of different species of the genus Miscanthus are increasingly covered in modern works of Russian (Dorogina et al., 2018, 2022; Gushchina et al., 2018; Berseneva et al., 2020; Kapustyanchik et al., 2020; Anisimov et al., 2021; Yakimenko et al., 2021) and foreign (Chae et al., 2014; Gifford et al., 2014) researchers. In them, M. sacchariflorus (Maxim.) Hack., M. sinensis Andersson and, especially, Miscanthus × giganteus J.M. Greef, Deuter ex Hodk., Renvoize are considered as sources of bioethanol and biocellulose. Currently, the Soranovsky variety is grown in the forest-steppe of Western Siberia. It was selected from the natural material M. sacchariflorus and is a valuable industrial crop (Potseluyev, Kapustyanchik, 2018).

M. sinensis and M. purpurascens Andersson are widely used as ornamental grasses and a set of varieties, including variegated ones, is already presented. M. sinensis is the most popular choice for decorating the banks of reservoirs, rock gardens, rockeries, mixborders, as a solitaire on the lawn and for creating decorative group plantings (Grechushkina- Sukhorukova, 2022). In natural conditions, M. sinensis and M. purpurascens grow in regions with a monsoon climate. Both species can be successfully grown in continental climates, according to long-term studies of growth and development rhythms, biomorphology and ontogenesis; however, M. sinensis has a higher adaptive potential (Dorogina et al., 2018). It is believed that in the harsh conditions of the foreststeppe of Western Siberia, all types of Miscanthus (fan grass) do not have time to form full-fledged seeds (Zueva, 2020).

The collection gene pool of the Central Siberian Botanical Garden of the Siberian Branch of the Russian Academy of Sciences (CSBG SB RAS, Western Siberia, Novosibirsk), is constantly replenished with new forms and varieties of Miscanthus from various habitats. When replenishing the collection with samples from different areas of Primorsky Krai, Russian Federation (RF), two forms (S1 and S2 – selected) of M. sinensis were selected by us. In the conditions of the continental climate of the forest-steppe of Western Siberia, they form viable seeds, and two generations (G1 and G2 – generation) were obtained from them. These forms were also characterized by accelerated rates of seasonal development and a more compact habitus. They form less vegetative mass and form generative organs earlier.

The purpose of this study: selection, biomorphological characterization of early flowering ornamental forms of Miscanthus sinensis and analysis of genetic polymorphism of the selected S1 and S2 forms and the obtained G1 and G2 generations using ISSR markers.

## Materials and methods

In 2017, M. sinensis seeds were collected from a coenopopulation on the Gamov Peninsula, Khasansky District, Primorsky Krai (42°58ʹ02″N; 131°20ʹ67″E), and sown in the ornamental plant collection plot of the CSBG SB RAS. As a result, only two plants with formed seeds were found in the forest-steppe conditions of Western Siberia (54°82ʹ15″N; 83°10ʹ46″E). Seeds formed on the earliest flowering shoots (more than 100 pieces were collected from five shoots). These plants, adapted to local climatic conditions, were used as initial forms (S1 and S2) to obtain subsequent G1 and G2 generations.

The experiments were conducted in two variants: in laboratory conditions in Petri dishes and when sowing in soil in a greenhouse. The dynamics of germination of the obtained seeds, the detection of the presence of a dormant period, the influence of environmental factors on their germination according to the laboratory-greenhouse-soil method of cultivation were estimated (Dyuryagina, 1982). As further studies showed, the seeds of the studied Miscanthus species were not in a state of deep dormancy, but in a forced dormancy

Option I. In the third ten-day period of February, M. sinensis seeds were placed on filter paper for germination in laboratory conditions, at room temperature (19–20 °C), in Petri dishes in 2–3 replicates, 50 seeds in each. A small number of replicates is due to the presence of an insignificant seed harvest. Such division into replicates allowed to comply with GOST (Methods for Testing…, 1973). The lighting was natural, filtered tap water was used as a humidifier. Regular observations of the condition of the seeds, the dynamics of germination, the nature of the growth of the primary root and primary shoot (primary leaf) were carried out.

Option II. M. sinensis seeds were sown in a stationary greenhouse in the second ten-day period of January in containers with a cell size of 2 × 2 cm, filled with a soil mixture of fertile soil, humus and sand. Three seeds were placed in a small depression in the soil (no more than 1 cm). They were lightly pressed down and carefully moistened by spraying, covered with glass or film, and not embedded in the soil. Germination energy and germination were recorded on the 7th and 21st day, respectively

**Fig. 1.**
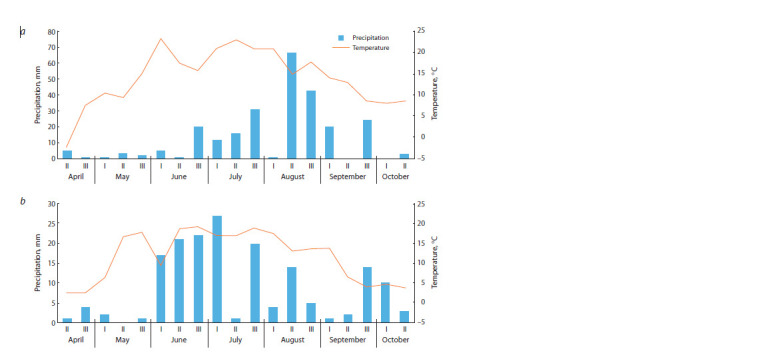
Hydrothermal conditions of the 2022 (a) and 2023 (b) growing seasons.

Hydrothermal conditions of the vegetation periods were calculated based on the data from the Ogurtsovo hydrometeorological station closest to the CSBG. The vegetation periods in 2022 and, especially, in 2023, were characterized by favorable meteorological conditions for flowering and fruiting of M. sinensis, similar in their dynamics to the hydrothermal indicators of the natural habitats of the species. In 2022, favorable hydrothermal conditions for plant growth and development developed from the second ten-day period of June and from the first of July. The average monthly air temperature in June and July was 17.3 and 18.9 °C, respectively (Fig. 1a). In 2023, the dry period in spring and the first half of summer was replaced by heavy precipitation in August (Fig. 1b).

To collect seeds in dry weather, panicles were cut after October 10. Based on the date of collection (ripening), flowering of this individual should have occurred in mid-August, and not in late July–early August

The species and forms of Miscanthus in the collection are studied as ornamental grasses. Therefore, the following measurements and calculations were carried out by us: shoot length (cm), panicle length (cm), number of leaves on the shoot (pcs.).

To study genetic variability, we used the method of electrophoresis of intermicrosatellite regions of genomic DNA (ISSR analysis). It is known that among anonymous methods of fragment analysis, it is the most convenient, sensitive and reproducible (Nei et al., 1979; Kashin et al., 2016). DNA from laboratory-dried leaves of M. sinensis was isolated using the STAB method (Doyle J.J., Doyle J.L., 1987).

To study the variability between the original forms and two generations, the ISSR primers we had tested for M. sacchariflorus (Poaceae) were used (Dorogina et al., 2018). In this work, we used the three most informative primers, 17899A, 17898B, UBS-857, characterized by a polymorphic and reproducible pattern (Dorogina et al., 2019, 2022).

PCR was performed on a C1000 amplifier (Bio-Rad, USA). The volume of the reaction mixture was 25 μl. It consisted of the following components: 1.5 units of Taq DNA polymerase (Medigen, Russia); 2.7 mM MgCl2, 0.8 mM ISSR primer (Medigen, Russia); DNA solution – 2 μl; water mQ H2O – 2 μl. The amplification consisted of several stages: DNA denaturation for 90 s at 94 °C and 35 cycles, each of which included 40 s at 94 °C, 45 s of primer annealing and 90 s at 42–56 °C. The duration of the final stage of prolongation of the nucleotide chain was 5 min at 72 °C.

Electrophoretic separation of amplification products in 1.5 % agarose gel in 1× TAE buffer at 4 V/cm was performed. For statistical data processing, the TREECON software packages (Van de Peer, De Wachter, 1997) were used. Each ISSR marker was considered dominant, genetic distances and the polymorphism level of each primer (P, %) was calculated according to M. Nei and W.H. Li (1979).

Molecular genetic formulas for passportization of the M. sinensis population have been compiled according to the principle proposed by A.A. Novikova et al. (2012). Based on amplified PCR DNA fragments, genetic passports in the form of genetic formulas are presented. The genetic formula contains information about the method used, primers, and amplified DNA fragments detected in the sample under study. Statistical processing of the results using the StatSoft EXCEL 6.0 and STATISTICA v.6.0 software packages has been performed. The reliability of differences in the variability of morphometric features has been assessed using nonparametric criteria (Mann–Whitney, U-test).

## Results

Morphological characteristic

The first full-fledged seeds of M. sinensis of local reproduc-tion were collected in 2020. They were set during free pollination of two plants (which formed several panicles with high-quality mature seeds). Later, plants of the first (G1) and second (G2) generations of the reproduction were grown from these seeds.

**Fig. 2.**
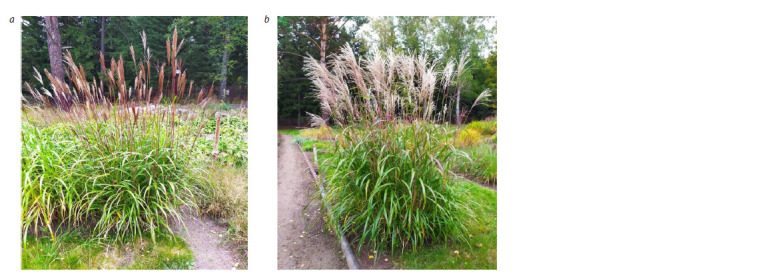
M. sinensis in CSBG SB RAS experimental plot. a – first generation (G1); b – second generation (G2).

The study of rhythmological and biomorphological features of the selected forms of M. sinensis showed the following. In the forest-steppe conditions of Western Siberia, early spring regrowth and earlier (at the end of July) flowering compared to other individuals obtained from the seeds of this sample were noted. In this regard, full-fledged seeds had time to set and ripen. Flowering of subsequent shoots has extended until October. The formation of compact bushes with shorter rhizomes is characteristic of these forms. Burgundy shoots and spreading panicles give the plants a special decorative effect (Fig. 2).

The straw is densely covered with ridges, belongs to the erect or semi-erectoid morphotype. Its height varies widely from 160 to 209 cm (Table 1). The leaf blades are elongated, linear (up to 70 cm), their width is from 0.6 to 1.2 cm. The edges of the leaf blades are very hard, the midrib is white. The panicle of this sample of M. sinensis is slightly drooping, characterized by 10–25 branches, 20–27 cm long (Table 1). The central axis of the panicle is shorter than the branches. Spikelets are paired. One of them has a short peduncle, and the other has a long one. The length of the spikelets reaches 4–7 mm. The spikelets at the base are pubescent with white hairs, their length can be equal to the length of the spikelet itself. These features create an additional decorative effect. We did not find any reliable differences in the variability of morphometric characteristics between G1 and G2 individuals.

**Table 1.**
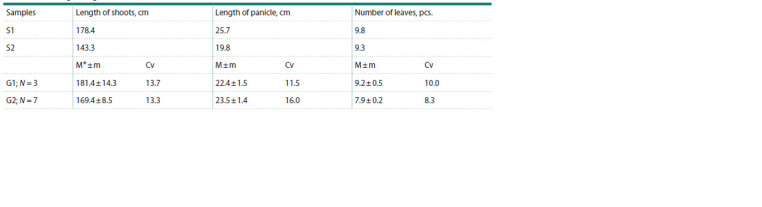
Morphometric indicators of Miscanthus sinensis shoots at the collection site of CSBG SB RAS at the end of the growing season * M – mean value; m – error of the mean; Cv – coefficient of variation (%).

The studied M. sinensis sample is resistant to local climatic conditions. These conditions correspond to the II–III frost resistance zones on the USDA scale (USDA Plant…, 2024). Therefore, it does not require winter shelter. During five years of introduction, M. sinensis was not affected by diseases and pests and was drought-resistant

A study of the biology of germination of two generations of M. sinensis seeds showed that when grown in a greenhouse, the beginning of germination in both generations was observed as early as day 3. On day 7, the germination energy of G2 seeds was 10 % higher than that of G1 seeds (Table 2). At this point, mass germination ended, and after 21 days, the germination of the observed samples increased by another 10 % in G1, and all the seeds of G2 germinated.

**Table 2.**
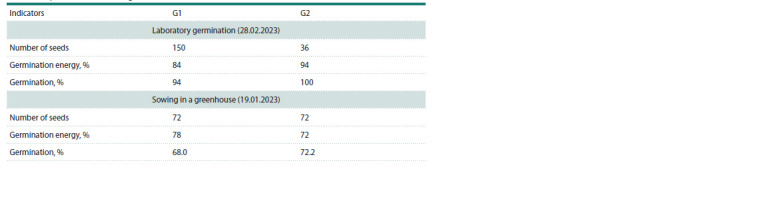
Germination of seeds of two generations of M. sinensis from reproduction seeds of CSBG SB RAS in laboratory conditions and in a greenhouse

Representatives of the genus Miscanthus in natural habitats begin vegetation at fairly high temperatures, therefore, for growing seedlings in a greenhouse (ontogenetic states: sprouts, juveniles and immatures), we have selected the optimal dates for sowing seeds.

When sowing seeds at the end of the second ten-day period of January, the sprouts grew and developed actively, their height on the 10th day reached 3 cm. The first true leaf in most G2 plants appeared on the 12th day after sowing. G1 sprouts lagged in development behind G2. The third true leaf in individual plants appeared on the 24th day of development. On the 50th day after sowing, the root system reached 6–8 cm in length, which exceeded the cell depth by 2–3 cm. At the same time, the leaves of the plants during the transition to the immature state, due to the small volume of cells for growth and development, noticeably turned yellow. Since there was still more than a month left before planting in open ground, the plants were transplanted into a larger container (7 × 7 cm). After transplanting, the immature plants began to actively develop and by the time of planting in open ground in the second ten-day period of May, their height varied from 9 to 31 cm.

Thus, it can be concluded that M. sinensis seedlings should be grown in greenhouse conditions, sowing in the second half of February. In this case, in the second half of May, the plants develop a branched fibrous root system, they enter the tillering phase, and they are ready for planting in open ground when favorable temperature conditions occur. Before planting in open ground, the plants need hardening. The seedlings were planted in a permanent place in warmed soil at a distance of 80–100 cm from each other. Thus, according to our observations, the seeds of the studied samples ripen well and do not require a period of post-harvest ripening, and laboratory conditions are more favorable for seed germination.

Marker analysis

**Fig. 3.**
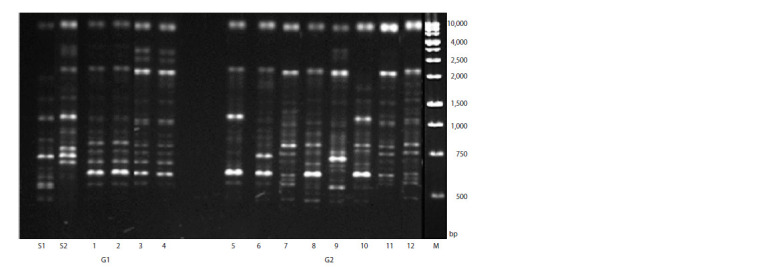
Electrophoregram of PCR products obtained by DNA amplification at the representatives of M. sinensis and ISSR primer UBC-857. S1, S2 – selected forms; tracks 1–4 – the first generation (G1), tracks 5–12 – the second generation (G2).

The study of variability in generations G1 and G2 using ISSR markers in G1 showed complete uniformity of amplification spectra. In generation G2, variability was observed, with five genotype variants revealed (Fig. 3).

On the electropherogram, in the region of less than 10,000 bp, a component present in each of the studied samples can be seen (Fig. 3). In all samples (except 10), in the region of 2,000–2,500 bp, a fragment from the S2 form is present. In the region of about 1,000 bp in samples 5 and 10 and in the region of slightly less than 750 bp in samples 6 and 9, fragments characteristic of both forms were present.

For the promising initial forms S1 and S2, selected by us, three markers were chosen for the purpose of compiling a genetic formula. They form the most polymorphic fragments (Table 3). The length of polymorphic fragments was from 300 to 3,000 bp. Using the (Ac)8GT primer, 2 and 4 unique fragments were detected, two of which were characterized by the greatest length (2,100 and 3,000 bp).

**Table 3.**
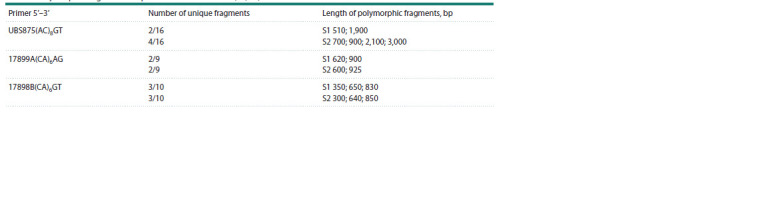
Polymorphic fragments unique to the selected forms (S1, S2) of M. sinensis Notе. Numerator – number of unique PCR fragments; denominator – total number of PCR fragments.

We have compiled genetic formulas for the selected forms S1 and S2 of M. sinensis using the identified unique molecular polymorphic fragments based on the genetic formulas for Rhododendron canadense (Novikova et al., 2012):

S1: ISSR/(AC)8GT-510,1900/(CA)6AG-620,900/(CA)6GT- 350,650,830 S2: ISSR/(AC)8GT-700,900,2100,3000/(CA)6AG-600,925/ (CA)6GT-300,640,850

## Discussion

In greenhouse conditions, we observed the germination of seeds of two generations of M. sinensis without preliminary treatment on the 3rd day, and on the 21st day, all seeds of G2 sprouted. The highest seed germination (98 and 88 %) was observed in M. sinensis and M. sacchariflorus grown in Korea at a temperature of 30 °C after scarification with 2 % NaOCl (Lee, et al., 2012). Polish scientists used sodium hypochlorite and commercial fertilizers to determine the cause of low germination of Miscanthus seeds and to search for methods to improve their quality (Orzeszko-Rywka, Rochalska, 2016).

The vegetation period in the geographical location of Primorsky Krai is quite long – 175–190 days, as our earlier analysis of the monsoon climate showed (Dorogina et al., 2019). Spring frosts are typical for the beginning of May, and autumn frosts, for the beginning of October. The spring-summer period is the driest. It is followed by a long summer-autumn overmoistening with up to 60 % of the annual precipitation rate.

Probably, the warm, relatively dry first half of summer in Primorsky Krai stimulates the intensive formation of generative, rather than vegetative organs of Miscanthus. Rains in the second half of summer and early autumn contribute to the filling of seeds and the formation of vegetative organs and buds for the next year’s renewal. As mentioned above, we observed similar dynamics in Novosibirsk in 2023 (Fig. 1). That is, the conditions of this growing season in Novosibirsk turned out to be close to natural.

According to the literature data, in the steppe zone of the Stavropol Territory (RF) during the vegetation periods of 2019–2021, 13 varieties of M. sinensis maintained rhythmic processes similar to natural ones (Grechushkina-Sukhorukova, 2022). Thus, the beginning of the growing season was from 12.04 to 17.04, and the growth processes in the flowering phase of early flowering varieties were from 5.08 to 12.08, mid-flowering – from 16.09 to 22.09, late flowering – from 12.10 to 18.10. The author showed that the dynamic indicators of the linear growth of generative shoots correlated with the sum of effective temperatures of the growing season: in 2019 r = 0.93–0.96; in 2020 r = 0.85–0.9; in 2021, r =0.9–0.92. These data are consistent with our results obtained in the forest-steppe of Western Siberia. The forms of M. sinensis that we selected had the ability to vegetate for a long time while maintaining decorative properties until the onset of winter dormancy of plants and can be successfully grown from seeds in continental climate conditions

As a result of the analysis of the obtained data for M. sinensis in the G2 population, we identified five genotype variants. G. Nie et al. (2014) showed that as a result of genotyping partially fertile hybrids in the hybrid population of M. sinen- sis, four genotypes were detected, and two of them were found in most plants. In Japan, tetraploid M. sacchariflorus and diploid M. sinensis are common, among which hybridization is observed (Tang et al., 2019).

However, M. sinensis is self-incompatible and has windborne pollen and seeds. According to our assumptions, this limits population differentiation. The degree of population differentiation using molecular markers for M. sinensis in individual areas of China has only been partially assessed (Chou et al., 2011; Swaminathan et al., 2012).

It is known from the literature that M. sinensis is a plant with a cross-pollination type (Mitros, et al., 2020). In general, interand intraspecific hybridization is typical for representatives of this genus, so it is characterized by rich genetic diversity and the presence of heterosis (Zhang et al., 2021). Genetic diversity is used to create Miscanthus hybrids. They can produce higher biomass yields and demonstrate better adaptability to various climatic conditions than their parent species (Clark et al., 2015). We did not conduct artificial hybridization. The two studied plants grew at a small distance from each other on the collection plot. G2 plants differed from the original (S1 and S2) selected forms in height, had more powerful leaves and stems. Therefore, we assumed the presence of hybrid plants as a result of spontaneous hybridization.

According to previous assumptions based on histochemical analysis of M. sinensis shoots, some specimens of this species can accumulate large amounts of lignin in dry, finished vegetation straws. This can complicate its industrial processing (Dorogina et al., 2019). Therefore, this species is more promising for the selection of ornamental forms

Most of the literature sources on various aspects of the study and practical application of species, forms, hybrids and varieties of Miscanthus note that within the framework of the collected gene pool, serious systematic clarifications are required (Greef et al., 1997; Nishiwaki et al., 2011; Gifford et al., 2014). Analysis of genetic diversity can also provide information on the origin and composition of individual lines (Xu et al., 2013, Chen et al., 2022).

The phenotypic and genetic variability we have discovered in M. sinensis allows us to select forms with various economically valuable traits for further genetic improvement and development of a variety with the desired traits. For example, interspecific hybrids between M. sacchariflorus and M. sinensis, such as Miscanthus × giganteus, are promising for obtaining biomass in culture in regions with a moderate climate. Such partially fertile hybrids are interesting for improving the biomass and quality characteristics of the Miscanthus species (Tamura et al., 2016; Chen et al., 2022).

Thus, breeding work with Miscanthus in severe climatic conditions moves to a fundamentally new level. It becomes possible to both test randomly selected forms (from natural habitats) and to work with a wide range of offspring from seeds of local reproductions of different generations. This expands the possibilities of selecting forms with different traits. So, further study of phenological rhythms, biology of seed germination, morphology, the analysis of genetic diversity and the differentiation of the population using molecular markers and selection of M. sinensis plant forms with valuable decorative and technical (technological) characteristics is promising.

## Conclusion

The forms of M. sinensis that we selected are highly decorative and resistant to introduction, produce viable seeds, and are promising for seed propagation and selection. They begin to bloom at the end of July and retain their decorative qualities until October. They do not require watering to maintain their decorative qualities during dry periods. M. sinensis grows successfully in open and shaded areas. It retains its decorative qualities in winter conditions and under snow. The offspring of the studied specimen pass all stages of ontogenesis in the conditions of Western Siberia and form viable seeds, and maintains its decorative effect from the end of July until October

The identified polymorphic fragments in M. sinensis can be used for identification and taxonomy, and unique molecular polymorphic fragments, which are sequences of a certain length, are the basis for the certification of populations, forms and lines that are promising for obtaining decorative forms of M. sinensis. Overall, our results in developing breeding programs will help with creating Miscanthus varieties with elite potential.

## Conflict of interest

The authors declare no conflict of interest.
